# Orbital adenoid cystic carcinoma treated by radiotherapy combined with anlotinib in a 13-year-old girl: A case report

**DOI:** 10.1097/MD.0000000000034544

**Published:** 2023-09-01

**Authors:** Xue Wu, Lili Qiao, Tiantian Tian, Yang Shu, Yingying Zhang

**Affiliations:** a Clinical Medical College of Jining Medical University, Jining, Shandong Province, China; b Department of Radiation Oncology, The First Affiliated Hospital of Shandong First Medical University, Changsha, China; c Shandong University of Traditional Chinese Medicine, Jining, Shandong Province, China; d Cheeloo College of Medicine, Shandong University Jining, Shandong Province, China.

**Keywords:** adenoid cystic carcinoma, anlotinib, case report, orbital, radiotherapy

## Abstract

**Rationale:**

Adenoid cystic carcinoma (ACC) of orbit is a very rare epithelial tumor, often originating from the lacrimal glands. At the same time, treatment options are currently limited, such as radiation, chemotherapy. We report a case of a patient treated with antirotinib combined with radiotherapy.

**Patient concerns:**

A 13-year-old girl was initially admitted with “left eye swelling for over half a year, 12 days after surgery for left orbital adenoid cystic carcinoma”. Initial swelling of the lateral upper eyelid of the left eye, with gradual enlargement and occasional pain.

**Diagnoses:**

Left orbital adenoid cystic carcinoma.

**Interventions:**

After diagnosis of orbital ACC, she underwent resection of the left orbital mass, and received 33 times of adjuvant radiotherapy, but brain metastases appeared later. She refused further treatment, and received 25 times of radiotherapy and anlotinib therapy after the disease progressed again.

**Outcomes:**

Now the patient has been followed up for 8 months, but no progress was found.

**Lessons:**

Based on this, we hypothesized that radiation therapy in combination with anlotinib is effective for ACC or ACC metastases.

## 1. Introduction

The lacrimal gland adenoid cystic carcinoma (LGACC) is rare and accounts for 1% of head and neck malignancies and usually originates in the lacrimal glands.^[1,2]^ The treatment of this disease is still limited to local treatments such as surgery and adjuvant radiotherapy. Distant metastatic disease often occurs even when the primary tumor can be controlled.^[3]^ Anlotinib is a novel small-molecule tyrosine kinase inhibitor that targets vascular endothelial growth factor receptor, fibroblast growth factor receptor, platelet-derived growth factor receptor, and c-Kit.^[4]^ In addition, it can not only inhibit tumor angiogenesis, but also inhibit tumor cell proliferation.^[5]^ In China, anlotinib has been approved for the treatment of non-small cell lung cancer, small cell lung cancer and soft tissue sarcoma.^[6,7]^ In this case report, we aim to report a case of anlotinib in the treatment of pediatric adenoid cystic carcinoma of the left orbital, which is rarely reported in the past.

## 2. Case report

**Figure 1.**
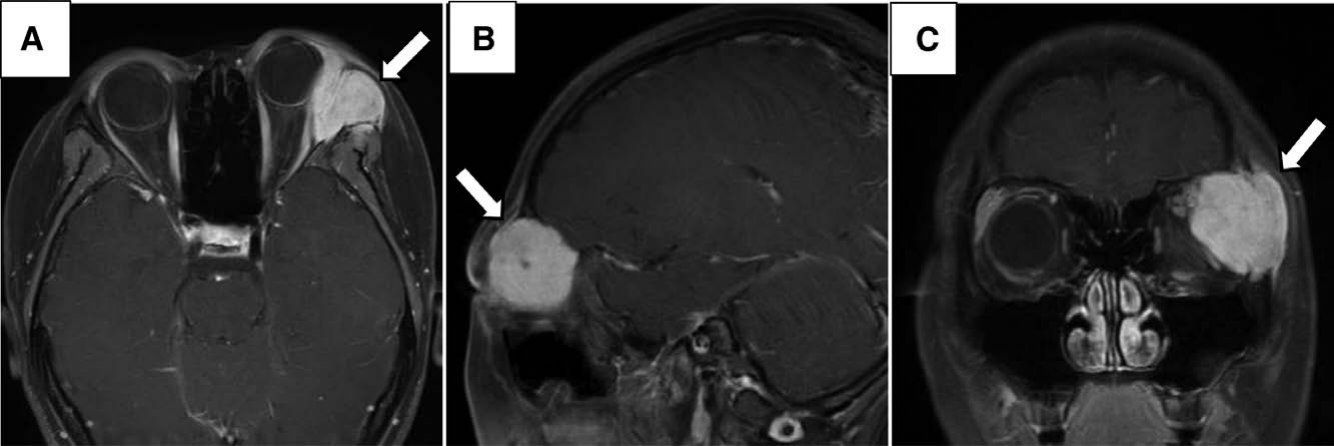
MRI from axial (A), sagittal (B), and coronal (C) views revealed an irregular mass in the left lacrimal gland region, about 3*3.2*3.2 cm in size. Left anterior process, adjacent external rectus muscle compression displacement, left orbital wall bone destruction. MRI = magnetic resonance imaging.

**Figure 2.**
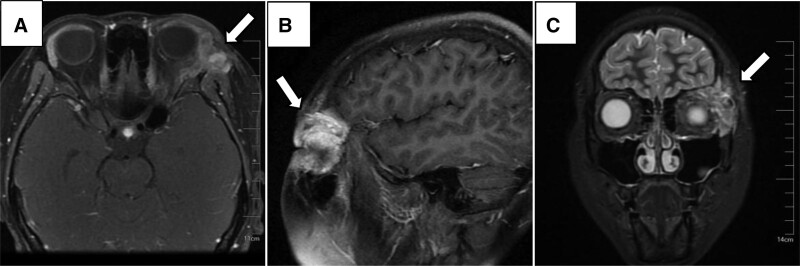
MRI from axial (A), sagittal (B), and coronal (C) views revealed an enhanced tumor on the lateral side of the left orbit involving the left orbital soft tissue, left eyelid, temporalis muscle, and left zygomatic arch. MRI = magnetic resonance imaging.

**Figure 3.**
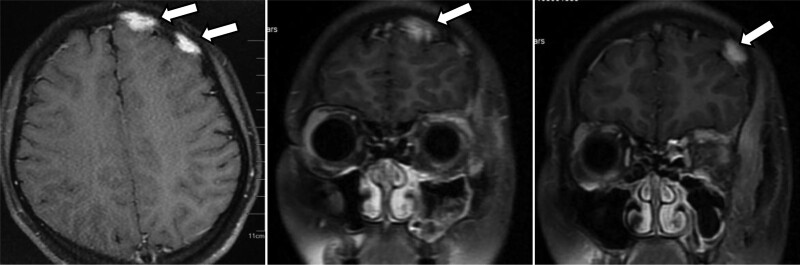
MRI showed two patchy isosignal foci on the left side of frontal bone, with sizes of 2.5 cm*1.1 cm and 1.8 cm*0.8 cm, respectively. The enhanced scan showed obvious enhancement. MRI = magnetic resonance imaging.

**Figure 4.**
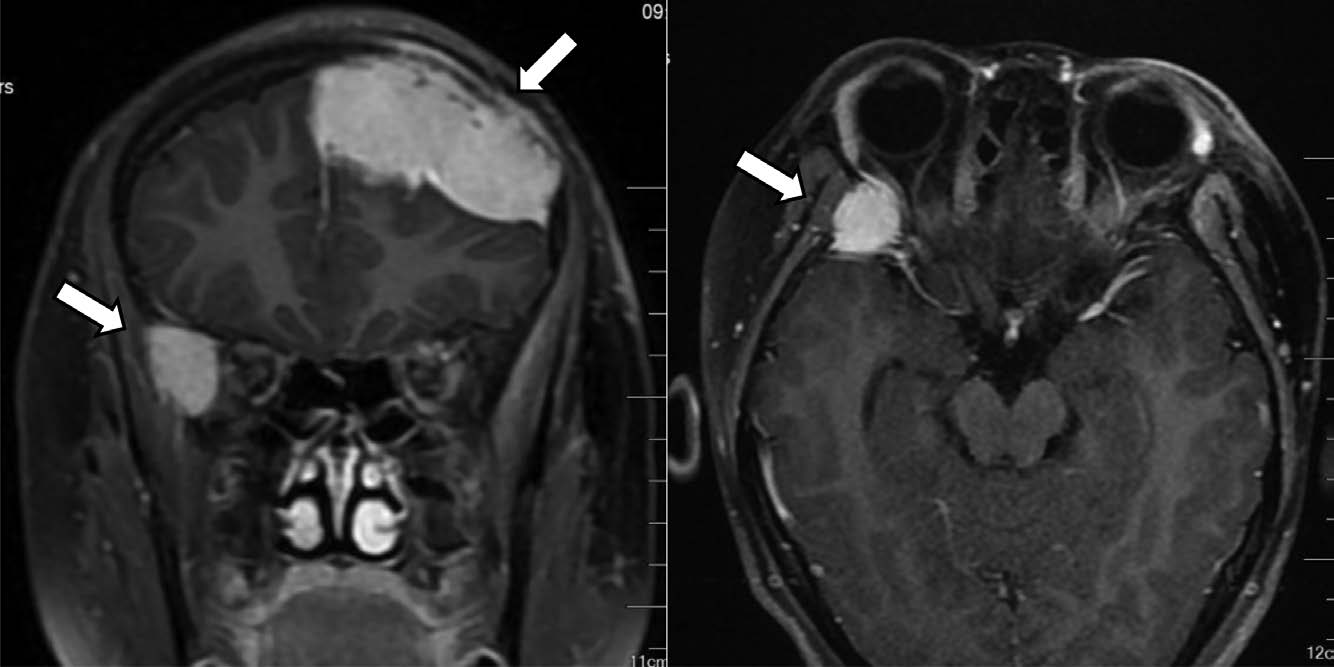
MRI showed that the enhancement focus of the left frontal bone was larger than before, with a cross section of 3.7 cm*3.1 cm. Nodular long T1 and long T2 abnormal signals were found in the right temporal region, ranging from 1.6 cm*2.2 cm, and the enhanced scan showed obvious enhancement. MRI = magnetic resonance imaging.

**Figure 5.**
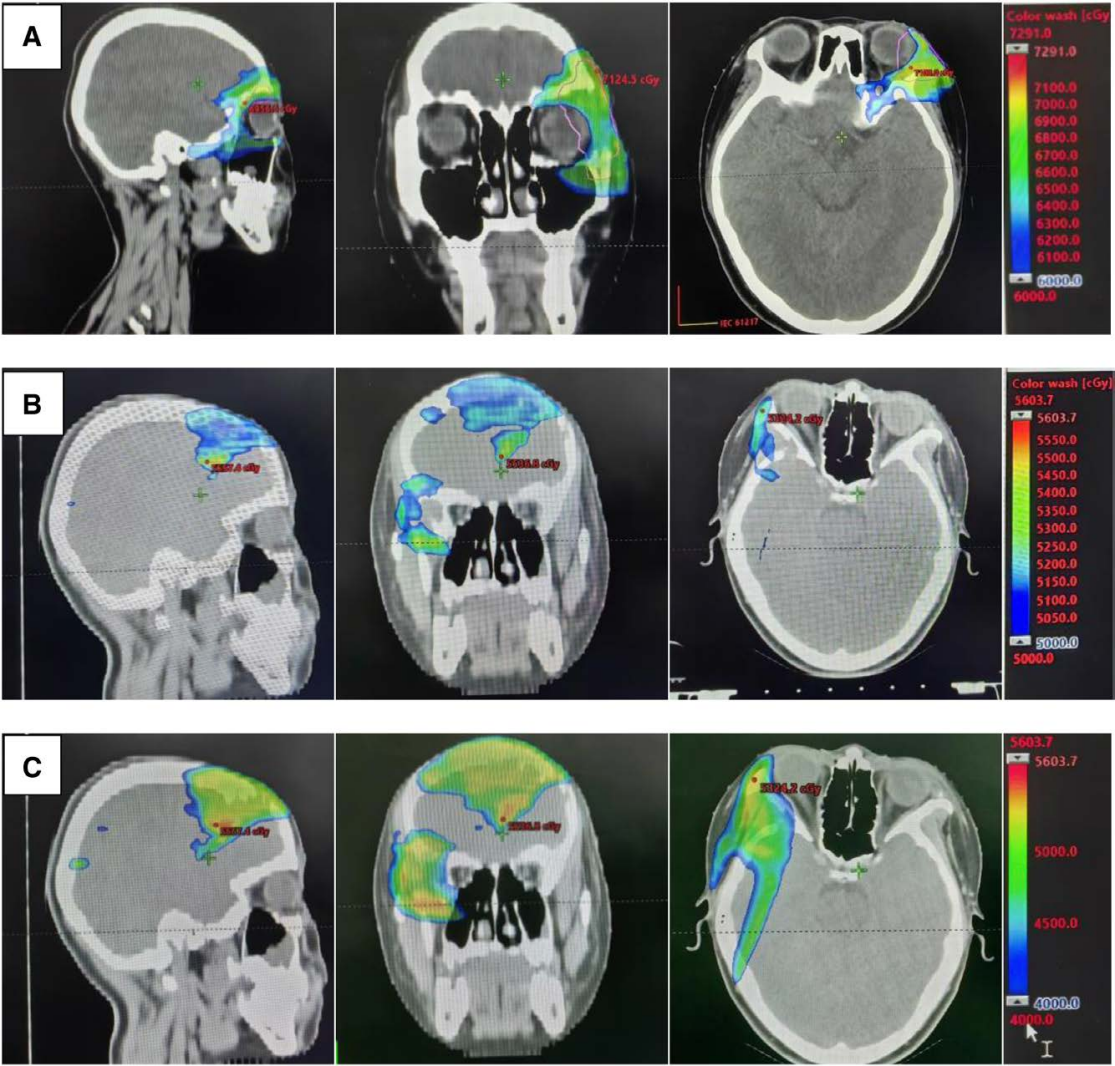
The radiotherapy protocol of the case.

**Figure 6.**
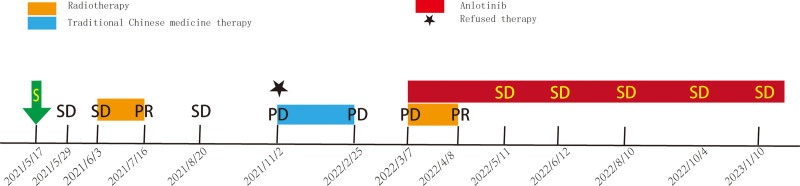
Timeline of all of the patient’s treatments. PD = progressive disease, PR = partial response, S = surgical treatment, SD = stable disease.

A 13-year-old girl presented to our radiation oncology department with a complaint of more than 1 year after left orbital surgery and 9 months after radiotherapy. In May 2021, she was admitted to Beijing Tong Ren Hospital. Magnetic resonance plain scan and enhanced scan of orbit were performed (Fig. 1) to consider lacrimal gland tumors. Intraorbital tumor resection was performed after excluding surgical contraindication. A mass about 5 cm*4 cm*3 cm in size was observed during the operation. The lateral orbital wall and supraorbital wall were involved, and the bone wall was damaged. During the operation, tissues with a size of 3.2 cm*2 cm*0.6 cm were sent for pathological examination, and the results were consistent with adenoid cystic carcinoma (ACC) with solid type about 85% and sieve type about 10%. Ki-67 is generally expressed strongly in the substantive type. This girl has Ki-67 proliferation index of about 50%. Besides, immunohistochemistry showed that carcinoembryonic antigen, epithelial membrane antigen, Vimentin, P53, CK7 and CD117 were positive in tumor cells. Unfortunately, the details of image and staging are unknown. The patient first visited our department in May, 2021, due to 12 days after surgery for adenoid cystic carcinoma of the left orbit. After completing the relevant examinations including head and neck magnetic resonance, chest and abdomen computed tomography and bone emission computed tomography, there are residual tumors on magnetic resonance imaging (MRI) (Fig. 2), without any metastasis. A Volume modulated intensity radiotherapy plan was employed to residual tumors at a total dose of 6600cGy and tumor bed including skull base canal with a dose of 6000cGy (33 fractions) (Fig. 5A). During radiation therapy, pain appeared in the left eye, and was diagnosed as dry eye after ophthalmic consultation, which was improved after symptomatic treatment. Three months after radiotherapy, MRI showed 2 new metastases in the frontal bone (Fig. 3), about 2.5*1.1 cm and 1.8*0.8 cm, respectively, and PET-CT was consistent with the findings of craniocerebral metastases. The patient refused any antitumor therapy and chose herbal as the alternative therapy for more than 3 months. By February 2022, new clinical and radiologic progression with the larger left frontal bone metastasis and new right maxillary sinuses metastasis established (Fig. 4). Her genetic test results were published in March, which show that MCL1 amplification and NOTCH1 mutation. After discussion in the multidisciplinary tumor board, she received palliative re-radiotherapy for metastases with scheme of 200 cGy per fraction, up to 25 fractions in total concomitantly with anlotinib targeted therapy (8 mg d1–14, q3w). Since that the patient was receiving radiation for the second time, the final dose of some parts (Fig. 5C) was less than originally planned (Fig. 5B) to protect her eye and lens. One month after the completion of radiotherapy, MRI and clinical examination were performed. Response-assessment was partial response (PR) with RESIST 3.0 criteria. Maintenance treatment with anlotinib regimens (8mg d1–14,q3w) were followed. During the follow-up, MRI showed a decreased signal and stable disease in terms of dimensions. The patient had a good tolerance with Kamofsky Performance Status 0 score. Figure 6 summarizes the patient’s entire course of treatment and disease status.

## 3. Discussion

ACC of orbit is a malignant epithelial tumor with a poor prognosis and often originates from the lacrimal glands. ACC accounts for 10% to 22% of salivary gland malignancies in the head and neck, and 1% of all head and neck malignancies.^[2]^ Carolina Emerick et al^[8]^ calculated that the 10-year disease-free survival rate for all patients with ACC was 29% to 50%, and the 10-year survival rate for LGACC was about 30%. Orbital ACC usually occurs in young and middle-aged people. ACC of orbit in children has been reported to be very rare. The malignant degree of these tumors may be lower in children, and the efficacy and prognosis of orbital ACC in children are better than those in young people.^[9]^

Early clinical manifestations are not typical, easy to misdiagnose or miss diagnosis, which diagnosis is often to the late stage. Pain is its main clinical feature, may also appear protrusion, double vision, vision loss and so on. In this case, the patient initially presented with swelling of the lateral upper eyelid of the left eye, which was gradually enlarged and accompanied by pain.

Orbital ACC most commonly originates from small salivary glands in the mouth and rarely extends into the skull.^[10]^ In addition, ACC has the characteristics of neurotropic invasion, and it is easy to metastasize to distant areas along the nerve infiltration, and the brain is a common site of its metastasis. In this case, however, it extends from the orbit along the periosteum into the skull. This has never been reported in the past.

MCL1 amplification and a Notch1 mutation was found in the patient’s genetic test. In previous studies, Notch1 mutations were associated with the invasiveness of LGACC.^[11]^ In previous studies, overexpression of Notch1 has shown significant reductions in overall survival and relapse-free survival in cancer patients.^[12,13]^ Shahzan Anjum et al^[13]^ investigated the relationship between Notch receptor expression and tumor aggressiveness in lacrimal ACC, and patients with Notch1 overexpression showed a greater likelihood of neuroaggression and a lower disease-free survival rate. Notch 1 plays an important role in cell growth and distant metastasis.^[14]^ Overexpression of MCL1 shows poor treatment resistance and prognosis in hematological malignancies. Currently, there are no studies on the correlation between MCL1 and ACC.^[15]^ On the other hand, ki-67 index of about 50% indicates that tumor recurrence and metastasis are more likely, which means the substantive type has a worse prognosis. P53 gene mutation is a high-risk factor for local recurrence after ACC surgery. All of these points indicate the high degree of malignancy and poor prognosis of the disease we reported on in this girl.

We report a case of orbital cystic adenoid carcinoma in a 13-year-old girl. In this case, the patient received surgery, radiotherapy and targeted therapy. After the patient received postoperative radiation alone, metastases of the left frontal bone were found at a review 3 months after the end of radiotherapy. This indicates that ACC is sensitive to radiotherapy, but the efficacy is not satisfactory. When the contralateral temporal metastasis recurred, the patient and her family decided to receive radiation therapy plus anlotinib. In addition, the dose of the second radiotherapy was obviously insufficient, especially for new metastases on the right side, but the therapeutic effect was still very obvious. This proves that anlotinib is effective in ACC, but whether the effect of anlotinib alone or whether it increases the effect of radiotherapy remains to be further explored. At present, the patient is still taking anlotinib. It has been 9 months, and she has been back to the hospital for reexamination for many times. Radiographic findings assessed her condition as PR. This once again indicates that the therapeutic effect of anlotinib on ACC of orbit in children is very significant. Additionally, the safety of anlotinib can also be guaranteed. When this girl first visited our department, she was 163cm tall and weighed 53KG. At her last admission, she was 170 cm tall and weighed 48kg. There was no delay in growth or puberty due to anlotinib. During all the treatments, she experienced orbital pain, head swelling pain, upper limb pain, hip pain and other symptoms, which were all improved after symptomatic treatment.

The MYB signaling pathway has been shown to up-regulate multiple target genes, including vascular endothelial growth factor a, fibroblast growth factor 2, and c-KIT.^[16]^ Anlotinib can be targeted to inhibit these targets.^[4]^ We suspect that this is a mechanism by which anlotinib is effective in ACC treatment. MYB can promote T cell development, and Notch signaling has been found to enhance this effect.^[17]^ In additional, Ning Su et al^[3]^ analyzed 19 patients with metastatic ACC treated with anlotinib and concluded that anlotinib had good antitumor activity and controllable toxicity against metastatic ACC. It can be seen from this case that anlotinib is effective in the treatment of orbital adenoid cystic carcinoma. However, the mechanism of anlotinib in the treatment of orbital adenoid cystic carcinoma may need further study.

In this case, a new metastatic tumor was found 5 months after radiotherapy. However, the patient has now been followed up for 9 months with multiple reviews of PR after reintroduction of radiotherapy plus anlotinib targeting therapy. We can’t define at this point whether it is the effect of anlotinib that enhances the radiation or whether it’s the effect of anlotinib itself. Yet, from this case, we can see that radiation therapy in combination with anlotinib is effective for ACC or ACC metastases.

## Author contributions

**Conceptualization:** Lili Qiao.

**Data curation:** Lili Qiao, Tiantian Tian, Yang Shu.

**Resources:** Yingying Zhang.

**Writing – original draft:** Xue Wu.

**Writing – review & editing:** Xue Wu.
